# Notes about Women

**Published:** 1896-06

**Authors:** 


### Notes About Women.

Dr. Eliza M. Mosher, of Brooklyn, was last January appointed a professor of hygiene in the University of Michigan and a dean of the literary department. The honor is one of the greatest ever paid to a professional woman. The place to which she was appointed had never before been held by a woman. Dr. Mosher was graduated from Ann Arbor in 1875. She has been superintendent of the Massachusetts Reformatory for Women and professor of physiology in Vassar College. She is a member of various medical societies and has made several valuable contributions to medical literature.

Dr. Sarah R. Adamson-Dolly, of Rochester, was the second woman in the United States to receive a full medical diploma, Dr. Elizabeth Blackwell being the first. Dr. Dolly graduated in 1851 as Dr. Adamson, marrying a little later Dr. Lester Dolly, the professor of anatomy and surgery in the school from which she graduated. Since 1872 Dr. Dolly has been a widow. Her son, Dr. Charles Dolly, is a prominent Philadelphia physician. Despite her advancing age Dr. Dolly still practises in Rochester.—*Abstract from Post-Express.*

Miss A. I. de Steiger, (*American Medical Review,*) a medical woman of London, was recently appointed third medical officer to the Essex County Lunatic Asylum, Brentwood. In order to make the appointment of a woman to this office regular it was necessary to alter the rules. England is coming on, despite the fact that her two leading universities still refuse to confer degrees upon women.

